# A Q-Learning-Based Distributed Energy-Efficient Routing Protocol in UASNs

**DOI:** 10.3390/e28030346

**Published:** 2026-03-19

**Authors:** Xuan Geng, Qingyuan Li, Xiaowei Pan, Fang Cao

**Affiliations:** Collage of Information Engineering, Shanghai Maritime University, Shanghai 201306, China; 202330310006@stu.shmtu.edu.cn (Q.L.); 202130310045@stu.shmtu.edu.cn (X.P.); fangcao@shmtu.edu.cn (F.C.)

**Keywords:** underwater acoustic sensor network, Q-learning, distributed routing

## Abstract

This paper proposes a Q-Learning-Based Distributed Energy-Efficient Routing (QDER) protocol for underwater acoustic sensor networks (UASNs). The routing problem is formulated as a Markov Decision Process (MDP) and a distributed Q-learning approach is proposed. Each sensor node is treated as an agent that independently selects its next-hop node based on a Q-table. The rewards function is designed that jointly considers node residual energy and depth information, enabling each node to learn an effective routing policy through distributed decision-making. Unlike centralized routing approaches that rely on extensive global information exchange, the proposed scheme allows nodes to make local decisions, thereby reducing communication overhead and energy consumption while maintaining efficient routing paths. In addition, link quality is designed in the reward to account for channel conditions, which improves the robustness of the routing strategy under noisy underwater acoustic environments. Simulation results demonstrate that the QDER achieves better system performance compared with Depth-Based Routing (DBR) and Deep Q-Network-Based Intelligent Routing (DQIR). Considering channel attenuation and noise, the proposed method with the link quality metric achieves improved network lifetime and energy efficiency. It also shows good robustness and adaptability under different signal-to-noise ratio (SNR) conditions.

## 1. Introduction

Underwater Acoustic Sensor Networks (UASNs) will be an important part of space–air–ground–sea integrated communication networks in future 6G networks [1]. They have gained significant attention for their diverse applications in oceanographic data collection, environmental monitoring, and underwater exploration [2,3,4]. Routing is particularly important in UASNs. The major challenges in UASN routing design include multipath fading, long propagation delay, low bandwidth, high bit error rate, and limited power. These challenges demand the development of energy-efficient and reliable routing protocols that can balance energy consumption, minimize latency, and maximize the network lifetime rate [5,6,7].

Many studies on underwater routing have been investigated in the last few years. Early studies mainly focused on Depth-Based Routing strategies due to their practicality. Hai et al. [8] proposed Depth-Based Routing (DBR) where a node selects the next forwarders with the shallowest depth. Although DBR provides a straightforward routing method, its flooding-based forwarding strategy leads to significant energy consumption. To improve energy efficiency in UASNs, Wahid et al. [9] introduced the Energy-Efficient Depth-Based Routing protocol (EEDBR), which determines forwarding decisions according to node depth and residual energy, thereby balancing energy consumption and time delay. Mhemed  et al. [10] proposed the Energy-Efficient Depth-Based Opportunistic Routing protocol (EEDOR), where routing decisions are made based on node depth and forwarding priority. Zhang et al. [11] developed the Energy-Efficient Probabilistic Depth-Based Routing protocol (EEPDBR) for underwater sensor networks. By jointly considering node depth, residual energy, and two-hop relay traffic load, EEPDBR improves the packet delivery ratio while reducing the average time delay. In addition, Rahman et al. [12] proposed the Energy-Efficient Cooperative Opportunistic Routing protocol (EECOR), in which the source node dynamically selects forwarding relays based on the energy consumption ratio and packet delivery probability. This design improves the packet delivery ratio while extending the network lifetime and reducing the end-to-end delay.

To adapt to the dynamic underwater communication environment, reinforcement learning (RL) has been widely investigated for routing design in recent years [13,14,15]. Because the agent in RL interacts with the surrounding environment by trial-and-error interactions, the RL-based methods exhibit self-adaptability to uncertain environments without a need for the predefined transmission model [16,17,18]. Zhou et al. [19] proposed a Q-learning-based Localization-Free Anypath Routing (QLFR) protocol. It calculates the Q-value according to the residual energy and depth of nodes, which makes a reasonable trade-off between depth and residual energy. Chen et al. [20] introduced a Q-learning based Multi-Hop Cooperative Routing (QMCR) protocol, which designs a routing protocol by use of Q-learning and combines a multi-hop collaboration strategy. It reduces computation complexity while achieving similar performance to the Artificial Fish-Swam Algorithm (AFSA) [21]. Gao et al. [22] also proposed a Q-learning-based routing algorithm for underwater wireless sensor networks (UWSNs), which jointly considers residual energy, transmission delay, and link success rate in reward design and introduces a priority-based holding time mechanism, achieving reduced delay and extended network lifetime compared with QLFR. An adaptive Deep Q-Network-Based Energy and Latency-Aware Routing (DQELR) protocol is presented in [23]. In this approach, a Deep Q-Network (DQN) is used to train the agent instead of Q-learning. The reward design considers both residual energy and depth, and a hybrid broadcast–unicast communication mechanism is adopted to reduce network overhead and improve energy efficiency. Our previous work studied a Deep Q-Network-Based Intelligent Routing protocol (DQIR), in which the agent trains the DQN to make decisions with global information [24]. It balances the energy consumption between nodes while minimizing the routing distances and performs lower energy consumption than that of DQELR.

To further improve robustness under time-varying underwater channels, reinforcement learning has also been combined with opportunistic routing in underwater acoustic networks. Zhang  et al. [25] proposed a routing protocol that combines Q-learning with opportunistic routing, where multiple relay nodes participate in the forwarding process collaboratively, thereby improving routing reliability and transmission efficiency in UWSNs. From the perspective of cross-layer design, Wang et al. [26] proposed an adaptive power-controlled Depth-Based Routing protocol to jointly optimize energy efficiency and energy balance through power control and next-hop selection, achieving an improved packet delivery performance. He et al. [27] proposed a cross-layer routing protocol based on channel quality information, allowing distributed nodes to improve link reliability and data delivery performance under a challenging underwater acoustic channel. The effectiveness of these RL-based routing schemes relies on the accuracy of channel state information and reliable link quality evaluation. At the physical layer, advanced signal processing techniques, such as fully connected neural network (FC-NN)-based channel estimation [28] and cepstrum transform techniques [29,30], can be employed to obtain channel state information and reduce the effect of multipath fading and noise. In addition, stable underwater communication links can be achieved by utilizing communication velocity features [31]. This physical-layer information can be further utilized in routing design to improve the reliability and efficiency of data transmission in underwater acoustic sensor networks.

Motivated by these observations, this paper proposes a Q-Learning-Based Distributed Energy-Efficient Routing (QDER) algorithm for underwater acoustic sensor networks. In the proposed framework, each forwarding node is treated as an independent agent, and the routing decision of each agent is formulated as a Markov Decision Process (MDP). A Q-learning-based method is adopted to solve the MDP, enabling nodes to learn effective forwarding strategies through interaction with the network environment. To guide the learning process, a reward function is designed by considering both node depth and residual energy, which helps balance energy consumption among nodes while maintaining efficient packet forwarding. Each node maintains its own Q-table and selects the next-hop node based on locally learned Q-values. In this way, routing decisions are made in a fully distributed manner without relying on global network information exchange. Considering the important role of link quality in underwater communication, the proposed method further uses the signal-to-noise ratio (SNR) at the receiving node as the metric of link quality in the reward design. The nodes are encouraged to select forwarding paths with more reliable communication links, thereby improving transmission robustness under dynamic underwater acoustic channels. The proposed scheme is suitable for large-scale underwater sensor networks with distributed nodes. Simulation results demonstrate that the method can reduce energy consumption and extend the network lifetime effectively, while maintaining reliable data delivery compared with conventional routing approaches. The main contributions of this paper are summarized as follows.

- A distributed reinforcement learning-based routing framework is developed for underwater acoustic sensor networks. The routing decision of each node is modeled as a Markov Decision Process (MDP), and a Q-learning algorithm is used to learn forwarding strategies in a fully distributed manner. By jointly considering node depth and residual energy in the reward design, the proposed method balances energy consumption among nodes while reducing the routing delay. The distributed design avoids the heavy information exchange required by centralized routing schemes and makes the algorithm more suitable for large-scale underwater sensor networks.
- Link-quality-aware routing is introduced by considering physical-layer channel information into the routing decision. In particular, the SNR at the receiving node is used as the link quality metric in the reward function. By utilizing this channel information during relay node selection, nodes are encouraged to choose links with better transmission conditions, thereby improving transmission robustness under dynamic underwater acoustic channels.

## 2. System Model and Reinforcement Learning

### 2.1. System Model

**Figure 1:**
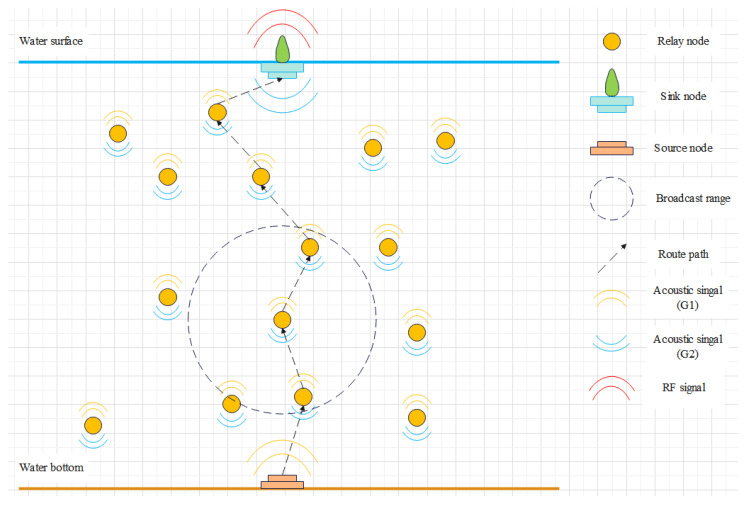
Network model.

Figure 1 illustrates the network model of UASNs, which consists of *N* nodes. Assume there are three types of nodes in the network, which are sink nodes, source nodes, and relay nodes, denoted by $N_{s}$, $N_{b}$, and $N_{f}$, respectively. The number of each type of nodes is denoted by $\left|N_{s}\right|=n_{s}$, $\left|N_{b}\right|=n_{b}$, and $\left|N_{f}\right|=n_{f}$, with the total number of nodes satisfying $N=n_{s}+n_{b}+n_{f}$. In this model, we assume $n_{s}=n_{b}=1$.

The source node is deployed at the water bottom to collect information and transmit signals, while relay nodes forward these signals to the sink node via multi-hop communication. Each relay node $N_{f_{i}}$ has its own candidate set $W_{N_{f_{i}}}$, from which it selects the next-hop node. The maximum transmission range of each node is denoted by *D*. At time slot *t*, the depth and residual energy of relay node $N_{f_{i}}$ are denoted by $d_{N_{f_{i}},t}$ and $e_{N_{f_{i}},t}$, respectively. In our model, each relay node selects only one node for data transmission at a given time, rather than transmitting the same data to multiple nodes simultaneously. The sink node is deployed at the water surface and is equipped with both acoustic and radio communication devices, allowing underwater acoustic signals to be converted into wireless radio signals for further transmission.

In this study, each nodes is equipped with two 180° semi-circular antennas for signal transmission. These antennas are installed horizontally on the nodes and oriented either upward or downward, denoted by $G_{1}$ and $G_{2}$, respectively. The source node is equipped with antenna $G_{1}$, while the sink node uses antenna $G_{2}$. Relay nodes are equipped with both $G_{1}$ and $G_{2}$, enabling them to receive and forward data during the communication.

### 2.2. Q-Learning

Reinforcement learning (RL) enables an agent to adapt to its environment through trial-and-error interactions. The learning process can be modeled as a Markov Decision Process (MDP), which is defined by a tuple 〈S,A,P,R〉, where *S*, *A*, *P*, and *R* denote the state space, action space, state transition probability, and reward function, respectively. At time slot *t*, the agent observes the current environmental state $s_{t}$ and selects an action $a_{t}$ according to a policy π. After executing action $a_{t}$, the environment transitions to a new state $s_{t+1}$ and returns a reward $r_{t}$. The objective of reinforcement learning is to obtain an optimal policy $\pi^{*}$ that maximizes the expected cumulative return. The policy is defined as the probability of selecting action $a_{t}$ given state $s_{t}$: $\pi \left(a_{t}|s_{t}\right)=P\left(A=a_{t}|S=s_{t}\right)$. The cumulative discounted return is defined as

(1) $$U_{t}=\sum_{k=0}^{\infty }\gamma^{k}r_{t+k+1},\;\;\gamma \in \left(0,1\right]$$

where γ denotes the discount factor, which controls the relative importance of future rewards. The action-value function $Q_{\pi }\left(s_{t},a_{t}\right)$ and the state-value function $V_{\pi }\left(s_{t}\right)$ are used to evaluate the expected return of taking an action and being in a state under policy π, respectively. These functions are defined as

(2) $$Q_{\pi }\left(s_{t},a_{t}\right)=E\left[U_{t}|S=s_{t},A=a_{t}\right]$$

(3) $$V_{\pi }\left(s_{t}\right)=E_{A}\left[Q_{\pi }\left(s_{t},A\right)\right]$$

The optimal action-value function $Q^{*}\left(s_{t},a_{t}\right)$ is

(4) $$Q^{*}\left(s_{t},a_{t}\right)=\max_{\pi }\;Q_{\pi }\left(s_{t},a_{t}\right)$$

According to Temporal Difference (TD) learning, the Q-value update is

(5) $$\begin{array}{c} Q\left(s_{t},a_{t}\right)\leftarrow \alpha \cdot \left[r_{t}+\gamma \cdot \max_{a^{\prime }}\;Q\left(s_{t+1},a^{\prime }\right)\right]+\\ \left(1-\alpha \right)\cdot Q\left(s_{t},a_{t}\right) \end{array}$$

where $\alpha \in \left(\left.0,1\right]\right.$ is the learning rate.

## 3. MDP Model

In many Q-learning-based underwater routing protocols, routing decisions are determined by a centralized node that collects global network information. This design usually requires frequent information exchange among nodes, which increases communication overhead and energy consumption. To reduce such overhead, a distributed Q-learning-based underwater routing protocol is proposed, where each node acts as an agent and makes routing decisions locally without centralized coordination.

### 3.1. Problem Formulation

To improve routing performance, we aim to design a routing scheme that balances energy consumption among nodes while minimizing the routing path. To achieve this goal, a metric $V_{i}$ is introduced to represent the status of the *i*-th forwarding node, which is defined as

(6) $$\begin{array}{c} V_{i}=\frac{\left|e_{N_{f_{i}},t}-e_{ave}\right|}{▵d} \end{array}$$

where $e_{N_{f_{i}},t}$ denotes the residual energy of the current node $N_{f_{i}}$, and $e_{ave}$ represents the average residual energy of node $N_{f_{i}}$ and its neighboring nodes. $\Delta d=|d_{N_{f_{i}},t}-d_{N_{f_{j}},t}|$ represents the depth difference between the current node $N_{f_{i}}$ and the next-hop node $N_{f_{j}}$. The objective is to find an optimal policy $\pi^{*}$ that minimizes $V_{i}$ for all forwarding nodes, which can be expressed as

(7) $$\pi^{*}=\mathrm{argmin}\sum_{i\in N_{f}\cup N_{b}}V_{i}$$

### 3.2. MDP Model Definition

The decision-making process is modeled as a Markov Decision Process (MDP), denoted by $\langle S,A,P,R\rangle$, and the elements are defined as follows.

#### 3.2.1. State Space(*S*)

Each forward node is treated as an independent agent executing its own Q-learning process. For node $N_{f_{i}}$, the current state at time slot *t* is defined as $s_{t}=N_{f_{i}}$. If node $N_{f_{i}}$ selects node $N_{f_{j}}$ as the next hop, the state transitions to $s_{t+1}=N_{f_{j}}$, and the Q-table is subsequently updated.

#### 3.2.2. Action Space(*A*)

At time slot *t*, the current node $N_{f_{i}}$ selects its next hop $N_{f_{j}}$ from its candidate set $W_{N_{f_{i}}}$. Thus, the action of $N_{f_{i}}$ is defined as $a_{t}=N_{f_{j}}$, meaning that node $N_{f_{i}}$ forwards the data packets to node $N_{f_{j}}$ through unicast communication.

#### 3.2.3. State Transition Probability(*P*)

$P\left(s_{t+1}|s_{t},a_{t}\right)$ represents the state transition probability from $s_{t}$ to $s_{t+1}$ after a node executes an action at time slot *t*. In this study, we initialize $P\left(s_{t+1}|s_{t},a_{t}\right)=1$.

#### 3.2.4. Reward(*R*)

To obtain the optimal policy, the routing decision should jointly account for path efficiency and energy balance. On the one hand, selecting a next-hop node with a smaller depth helps the packet move closer to the sink, thereby reducing the number of forwarding hops and the total propagation delay. On the other hand, the residual energy of candidate nodes should also be considered to avoid the frequent use of low-energy nodes and to improve energy balance in the network. Based on these considerations, the reward function is designed as follows

(8) $$\begin{array}{c} r_{t}=\beta_{1}\cdot e_{N_{f_{i}},t}+\beta_{2}\cdot d_{N_{f_{i}},t} \end{array}$$

where $\beta_{1}$ and $\beta_{2}$ are weighting factors for energy and depth, respectively, with $\beta_{1},\beta_{2}\in \left(0,1\right]$ and $\beta_{1}+\beta_{2}=1$. By adjusting $\beta_{1}$ and $\beta_{2}$, we can choose which factor plays the main role in routing selection. A higher $\beta_{1}$ makes nodes more sensitive to energy, and selecting relay nodes with higher residual energy balances the energy consumption of the network. On the contrary, a higher $\beta_{2}$ makes nodes more sensitive to depth and selecting shallower neighbors as the next hop nodes, which reduces the number of hops, and thus reduces the propagation delay.

In practical underwater environments, acoustic propagation loss and noise can significantly affect transmission performance. To account for these channel effects in the routing decision, the reward function is further extended to include link quality. In this study, link quality is measured using the SNR at the receiving node, which is defined as

(9) $$\begin{array}{c} {\tilde{r}}_{t}=\beta_{1}\cdot e_{N_{f_{i}},t}+\beta_{2}\cdot d_{N_{f_{i}},t}+q_{N_{f_{i,j}},t} \end{array}$$

where $q_{N_{f_{i,j}},t}$ is the indicator of link quality between node $N_{f_{i}}$ and $N_{f_{i}}$, and it is denoted by

(10) $$\begin{array}{c} q_{N_{f_{i,j},t}}=\min\left\{1,\max(0,\frac{\zeta_{N_{f_{i,j},t}}-\zeta_{\min,t}}{\zeta_{\max,t}-\zeta_{\min,t}})\right\} \end{array}$$

where $\zeta_{N_{f_{i,j},t}}$ represents the SNR of the received signal transmitted from $N_{f_{i}}$ to $N_{f_{j}}$. The $\zeta_{\max,t}$ and $\zeta_{\min,t}$ denote the maximum and minimum SNR predefined by the nodes, respectively. By introducing the link quality factor into the reward function, the channel conditions are considered in the routing decision. This design encourages nodes to select receivers with higher SNR links, thereby improving transmission quality and the successful packet delivery rate.

### 3.3. Distributed Q-Table

**Figure 2:**
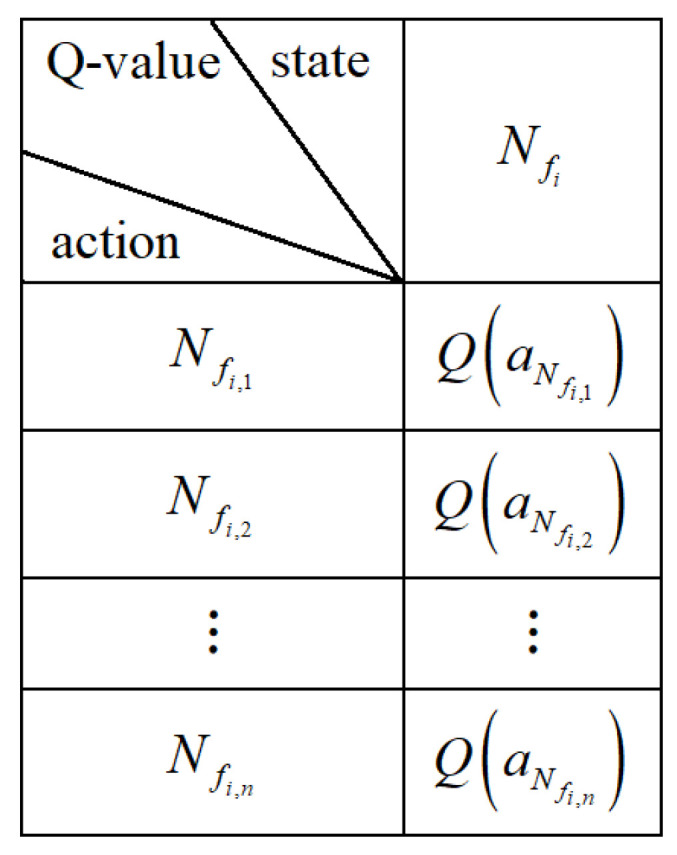
Distributed Q-table of $N_{f_{i}}$.

Using local information, each node makes routing decisions independently. In the proposed framework, the node itself is defined as the state, as described in Section 3.2. Accordingly, the Q-value associated with node $N_{f_{i}}$ is denoted as $Q(a_{N_{f_{i}}})$. Each node maintains its own Q-table, referred to as a distributed Q-table. Figure 2 shows the Q-table of node $N_{f_{i}}$, where $N_{f_{i,n}}$ represents the *n*-th candidate node in the candidate set $W_{N_{f_{i}}}$. The value $Q(a_{N_{f_{i,n}}})$ corresponds to the Q-value when $N_{f_{i,n}}$ is selected as the next-hop node. With this distributed design, each node only stores the current state and the Q-values associated with its candidate neighbors, without requiring global network information. As a result, memory overhead is reduced and energy consumption is reduced.

## 4. QDER Protocol

### 4.1. Packet Format Definition

**Figure 3:**
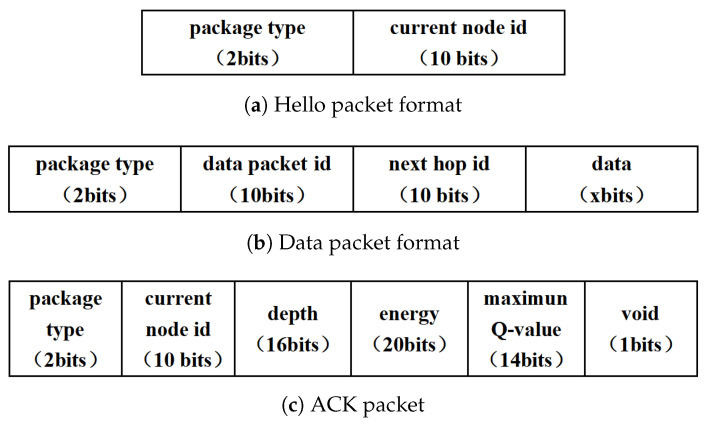
Packet format.

We define three types of data packet formats, as illustrated in Figure 3a–c. The structure of the Hello packet is shown in Figure 3a. The sink node broadcasts a Hello packet, which is subsequently forwarded by all nodes in the network. Hello packets are transmitted in two scenarios. First, the initialization for the entire network to establish the neighbor table for each node is performed. Second, the sink regularly sends the Hello packet at fixed time intervals to update the neighbor table of each node.

During data transmission, the relay node $N_{f_{i}}$ forwards the data packet to the selected next-hop node, such as $N_{f_{j}}$. The structure of the data packet is shown in Figure 3b. It contains the ID of the current node ($id_{N_{f_{i}}}$), the ID of the next-hop node ($id_{N_{f_{i}},N_{f_{j}}}$), and the transmission data.

After receiving the data packet, node $N_{f_{j}}$ continues forwarding the packet using antenna $G_{1}$. At the same time, it sends an ACK (Acknowledgment) packet to the previous node through antenna $G_{2}$ as feedback. The structure of the ACK packet is illustrated in Figure 3c. It includes $id_{N_{f_{j}}}$, $d_{N_{f_{j}},t}$, $e_{N_{f_{j}},t}$, and $Q_{\max}\left(a_{N_{f_{k}}}\right)$, where k≠j for node $N_{f_{j}}$. Additionally, the *void* indicates whether node $N_{f_{j}}$ is a void node. By default, this field is set to *False*.

### 4.2. Routing Algorithm

#### 4.2.1. Network Initialization

**Figure 4:**
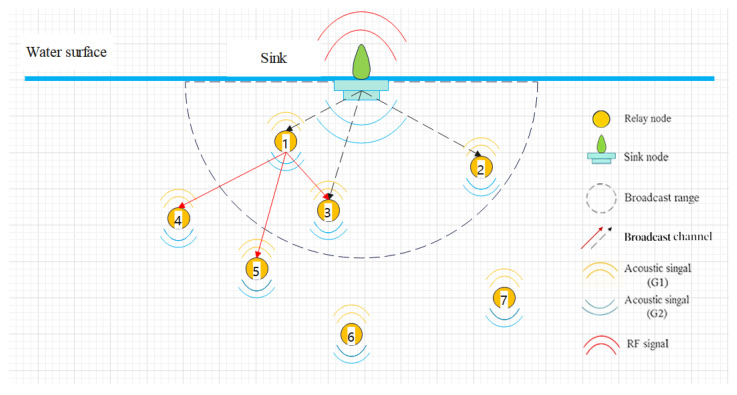
Network initialization by Hello packet.

The purpose of the network initialization is to enable each node to discover its neighboring nodes. Figure 4 illustrates the initialization process with seven nodes as an example. To start this process, the sink node generates and broadcasts a Hello packet when the network is first deployed. Let $▵\tilde{d}=\left|d_{N_{f_{i}},t}-d_{N_{f_{j}},t}\right|$ denote the distance difference between nodes $N_{f_{i}}$ and $N_{f_{j}}$. If $▵\tilde{d}<D$, node $N_{f_{j}}$ will receive the Hello packet. Therefore, nodes $N_{f_{1}}$, $N_{f_{2}}$, and $N_{f_{3}}$ receive the Hello packet from the sink node and add the sink to their candidate sets $W_{N_{f_{1}}}$, $W_{N_{f_{2}}}$, and $W_{N_{f_{3}}}$, respectively. Subsequently, node $N_{f_{1}}$ broadcasts Hello packets within its transmission range *D*. As a result, nodes $N_{f_{3}}$, $N_{f_{4}}$, and $N_{f_{5}}$ receive the packets. Since node $N_{f_{1}}$ is located at a shallower depth than these nodes, it is added to their candidate sets. In this way, each node builds its candidate set based on the received Hello packets and the depth information of the neighboring nodes. After the candidate sets are established, the Q-tables are initialized. The Q-values corresponding to candidate nodes are initialized as $Q(a_{N_{f_{i}}})=0$, while the Q-value of the sink node is set to $Q(a_{N_{s}})=100$. The initialization procedure continues until all nodes complete the construction of their respective Q-tables.

#### 4.2.2. Pre-Training

After network initialization, pre-training is performed before online transmission begins. In this stage, nodes exchange short training packets and update their Q-tables based on the routing selection. The purpose of this stage is to provide pre-trained Q-tables so that nodes can obtain routing knowledge. During this stage, each node updates its Q-table using an exploration strategy. We define a selection factor ε∈[0,1] and a threshold $\varepsilon_{0}=\mu \cdot \frac{\tilde{t}}{T}$, where μ∈[0,1] is a constant and $\tilde{t}$ and *T* denote the current training step and the maximum number of training steps, respectively. If node $N_{f_{i}}$ generates a random number ε such that $\varepsilon \geq \varepsilon_{0}$, it randomly selects a node from its candidate set $W_{N_{f_{i}}}$ as the next hop. Otherwise, $N_{f_{i}}$ selects node $N_{f_{j}}$ with the maximum Q-value $Q_{\max}\left(a_{N_{f_{j}}}\right)$ as the next-hop node. Afterward, node $N_{f_{i}}$ updates the Q-value $Q(a_{N_{f_{j}}})$ according to the ACK packet received from node $N_{f_{j}}$.

**Figure 5:**
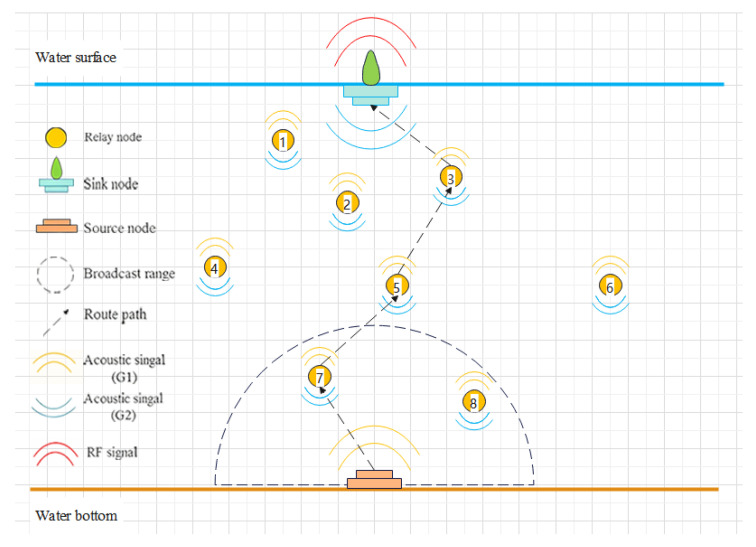
Next hop selection example.

**Figure 6:**
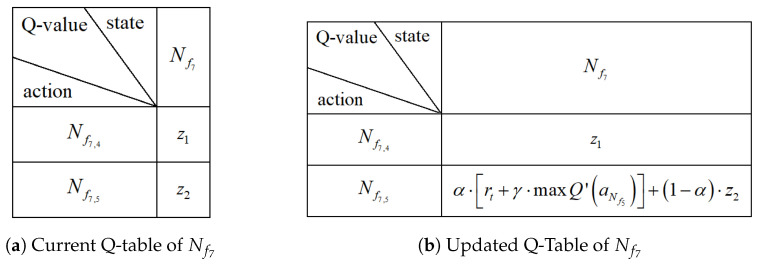
Q-table update example.

We take an example to illustrate the Q-table update. As shown in Figure 5, the current node $N_{f_{7}}$ attempts to select its next hop, and its current Q-table is presented in Figure 6a. Assume that $\varepsilon <\varepsilon_{0}$. In this case, node $N_{f_{7}}$ selects the node with the maximum Q-value from its Q-table. Suppose that $z_{2}>z_{1}$ in this example, i.e., $Q\left(a_{N_{f_{5}}}\right)>Q\left(a_{N_{f_{4}}}\right)$, as shown in Figure 6a. Therefore, node $N_{f_{7}}$ selects $N_{f_{5}}$ as the next-hop node and transmits a short data packet for Q-table training. After receiving the packet, node $N_{f_{5}}$ sends an ACK packet, as defined in Figure 3c, which contains the maximum Q-value in its Q-table. Based on this information, node $N_{f_{7}}$ updates the value of $Q(a_{N_{f_{5}}})$ in its own Q-table, while the value of $Q(a_{N_{f_{4}}})$ remains unchanged, as illustrated in Figure 6b. This process completes the Q-table update for node $N_{f_{7}}$ with one hop. As the process continues, the Q-tables of all nodes eventually converge.

Note that, during the initial phase of pre-training, relay nodes tend to select the next hop randomly because insufficient training data are available. As the pre-training continues, nodes increasingly prefer selecting the node with the maximum Q-value, $Q_{\max}\left(a_{N_{f_{j}}}\right)$, as the next-hop node in order to optimize the Q-values. At the end of the pre-training stage, when $\tilde{t}=T$, the threshold becomes $\varepsilon_{0}=\mu$.

#### 4.2.3. Online Transmission

After all nodes obtain their stable Q-tables, they will transmit the data packets online. The current node $N_{f_{i}}$ selects the node $N_{f_{j}}$ with the maximum Q-value, $Q_{\max}\left(a_{N_{f_{j}}}\right)$, from its candidate set $W_{N_{f_{i}}}$ as the next-hop node. All nodes within the transmission range *D* of node $N_{f_{i}}$ receive the data packet and identify the next-hop ID ($id_{N_{f_{i}},N_{f_{j}}}$). If $id_{N_{f_{i}},N_{f_{j}}}\neq id_{N_{f_{j}}}$, the data packet will be discarded. Otherwise, node $N_{f_{j}}$ forwards the data packet and sends an ACK packet with a *False* void flag to node $N_{f_{i}}$. Subsequently, node $N_{f_{i}}$ updates the Q-value $Q(a_{N_{f_{j}}})$ in its Q-table during online operation. If $N_{f_{i}}$ cannot receive ACK successfully, the transmission fails. Two reasons lead to unsuccessful reception, which are

(1) The next hop node $N_{f_{j}}$ did not receive the data packet successfully.
(2) The ACK failed to transmit in the feedback link.

For these two situations, the source node will resend the data packet. For the first situation, the node $N_{f_{j}}$ repeats the receiving process as mentioned above. For the second situation, the $N_{f_{j}}$ only needs to feedback ACK again and does not need to forward the data packets this time. If the $N_{f_{i}}$ cannot receive the ACK again, it will mark $N_{f_{j}}$ as a void node and disable the $N_{f_{j}}$ until the next Hello packet arrives.

We summarized the QDER protocol in Algorithm 1.
**Algorithm 1** QDER Protocol.  1:Initialize: Q-table, candidate set $W_{N_{f_{i}}}$, retransmission limit $p_{re}$, Hello period $p_{t}$, threshold $\varepsilon_{0}$  2:**for** each episode **do**  3:      **for** each timeslot t=1,…,T **do**  4:            Initialize retransmission counter num=0  5:            Select next hop $N_{f_{j}}$: ϵ-greedy from $W_{N_{f_{i}}}$ with probability $\varepsilon_{0}$  6:            Transmit packet to $N_{f_{j}}$; num←num+1  7:            **if** ID verification fails **then**  8:                  $N_{f_{j}}$ drops packet  9:            **else**10:                  $N_{f_{j}}$ forwards packet and sends ACK11:            **end if**12:            **if** no ACK received and $num<p_{re}$ **then**13:                  Re-select $N_{f_{j}}$ and go to step 614:            **else if** no ACK received and $num=p_{re}$ **then**15:                  Select alternative next hop and go to step 616:            **end if**17:            **if** $N_{f_{j}}$ is void **then**18:                  Disable $N_{f_{j}}$19:            **end if**20:            Update Q-table of $N_{f_{i}}$21:            **if** $t\;\mathrm{mod}\;p_{t}=0$ **then**22:                  $N_{s}$ broadcasts Hello packet23:            **end if**24:      **end for**25:**end for**

## 5. Simulation Results

### 5.1. Parameter and Metric Definitions

The underwater environment and routing protocol are simulated in this section. Suppose the underwater sensor nodes are deployed randomly in a three-dimension space within 500 × 500 × 500 $m^{3}$. One source node is deployed to the bottom of the water, and one sink stays on the water surface. The transmission rate is set as 5000 bps, and the packet format is given in Figure 3. The loop number is set to be $5\times 10^{3}$ per simulation trail. The hyper-parameters of Q-learning, such as the discount factor, learning rate, and ϵ-greedy factor are γ=0.5, α=0.9, and μ=0.1. A relatively high learning rate allows nodes to rapidly adapt to dynamic underwater channel conditions and topology variations, while a moderate discount factor enables the algorithm to consider both immediate and future rewards during routing decisions. Other parameters and symbol definitions are shown in Table 1 and Table 2, respectively. We note that the two weights of reward are set to be $\beta_{1}=0.8,\;\beta_{2}=0.2$ in the simulation, which is primarily to account for the impact of energy. However, it can be flexibly adjusted according to the relative importance of energy and depth in different scenarios.

In addition to parameter definitions, the performance evaluation metrics in the simulation are defined as follows.

(1) The total energy consumption *E*

The total energy consumption is defined as the sum of the energy consumed by all sensor nodes in the network during their working time, which is

(11) $$E=\sum_{i=1}^{n_{f}}E_{N_{f_{i}}}$$

The $E_{N_{f_{i}}}$ is computed by

(12) $$\begin{array}{cc} E_{N_{f_{i}}} & =\left(p_{s}\cdot T_{s_{i}}+p_{r}\cdot T_{r_{i}}\right)\cdot M_{data}\\ \; & \;+\left[T_{a_{i}}-\left(T_{s_{i}}+T_{r_{i}}\right)\cdot M_{data}\right]\cdot p_{w} \end{array}$$

in which the definitions of the parameters are listed in Table 2.

(2) Energy consumption ratio $E_{R}$

The energy consumption ratio represents the ratio of the total network energy consumption to the total initial energy of the network, which can be expressed as

(13) $$E_{R}=\frac{E}{n_{f}\cdot e_{ini}}$$

The calculation for energy consumption variance is

(14) $$E_{v}=\frac{1}{n_{f}}\sum_{i=1}^{n_{f}}{\left(E_{N_{f_{i}}}-E_{ave}\right)}^{2}$$

(3) End-to-end time delay

The end-to-end time delay is the time duration from a data packet transmitted at the source node to its successful reception at the sink via multi-hop relaying.

(4) Information exchange delay $TDC_{m}$

We defined an information exchange delay here to denote the time consumed by information exchange between nodes in the process of network forwarding the *m*-th data packet from a source node to the sink. The exchanged information includes ACK, node identifiers, packet-type indicators, and other related information required for routing selection. Accordingly, the information exchange delay for the *m*-th packet is

(15) $$TDC_{m}=\sum_{i=1}^{n_{f}}\sum_{j=1}^{n_{f}}T_{d_{i,j}},i\neq j$$

The average information exchange delay $TDC_{ave}$ is denoted by

(16) $$TDC_{ave}=\frac{\sum_{m=1}^{M_{data}}TDC_{m}}{M_{data}}$$

(5) Network lifetime

The network lifetime is defined as the time duration from the start of network operation to the time when the first node in the network exhausts its energy.

### 5.2. Performance Evaluation

In this section, we evaluate the routing performance proposed by this paper. We assume that the physical channels are error-free in the simulation, which may be achieved by strong forward channel correction coding and equalizer. Therefore, we focus on how routing decisions influence the system performance.

#### 5.2.1. Convergence of Q-Value

**Figure 7:**
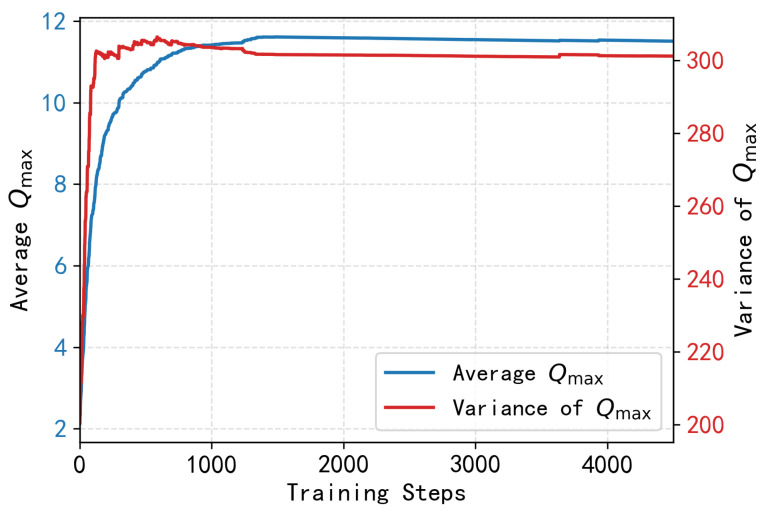
$Q_{max}$ convergence curves.

We evaluate the convergence performance for the Q-value first. In Figure 7, we draw an average $Q_{max}$ convergence curve with the training steps. It shows that the average $Q_{max}$ increases rapidly during the first 800 training steps and then stays stable after 1000 steps. The variance curve stabilizes at approximately step 900 and remains at a low level thereafter, indicating that the local Q-tables of all nodes have converged to a consistent state and thus the distributed learning process has reached a stable state.

#### 5.2.2. Performance of Energy Consumption

**Figure 8:**
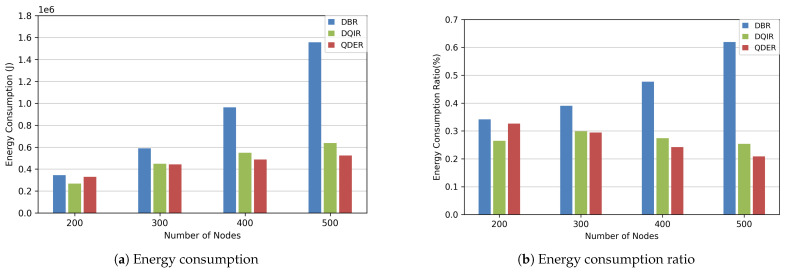
Energy consumption/ratio.

We compare the energy consumption of the proposed protocol (QDER) with DBR [8] and DQIR [24] in this section. Figure 8a,b show the total energy consumption results. As the node number increases, all protocols experience an increase in energy consumption. The DBR needs more repeated transmission, resulting in a significant increase in energy consumption. However, QDER is able to determine subsequent hop nodes, which reduces energy loss caused by repeated transmission. From Figure 8b, the energy consumption ratio of QDER and DQIR decreases with the increase in node numbers. In the case of fewer nodes, DQIR has a better energy consumption rate than QDER. However, as the number of nodes increases, DQIR requires more information exchange, resulting in a higher energy consumption ratio. These results demonstrate that the QDER can effectively select the optimal route in dense networks and use less information exchange, thereby effectively reducing energy consumption.

**Figure 9:**
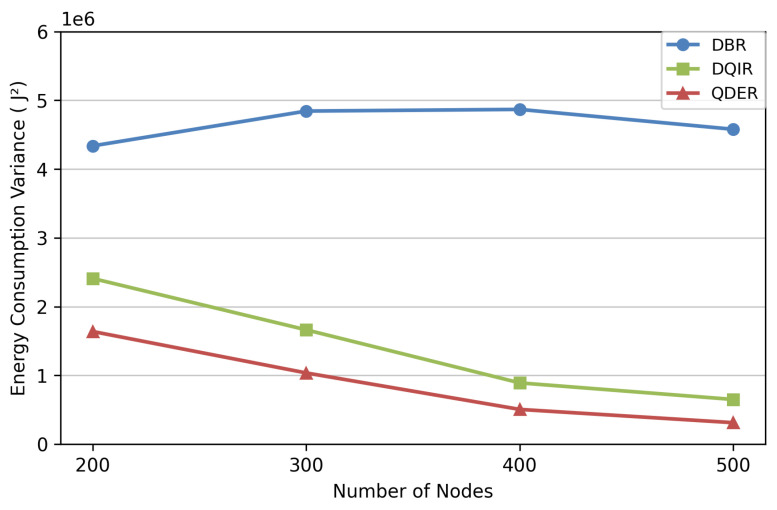
Variance in energy consumption.

Figure 9 shows the variance in energy consumption. The energy consumption of DBR is imbalanced, and therefore has the largest variance. The energy consumption of QDER and DQIR is relatively balanced. The information exchange of QDER is less than DQIR; therefore, the variance in energy consumption is also less than that of DQIR.

#### 5.2.3. Delay and Network Lifetime

**Figure 10:**
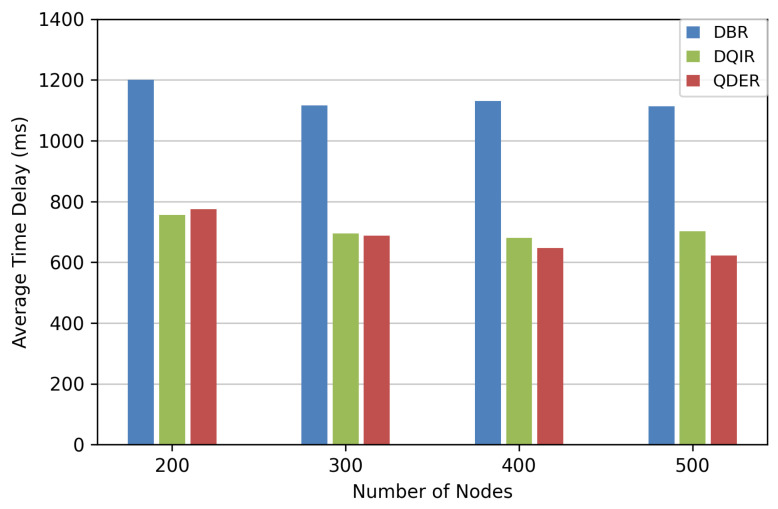
Average end-to-end time delay.

We evaluate the time delay and network lifetime in this section. Figure 10 illustrates the average end-to-end time delay of data transmission from the source node to the sink node. As the number of nodes increases, the average end-to-end time delays of QDER and DQIR are much shorter than that of DBR. Our algorithm significantly reduces the time delay by about 35% compared with DBR, and its performance is very close to DQIR.

**Figure 11:**
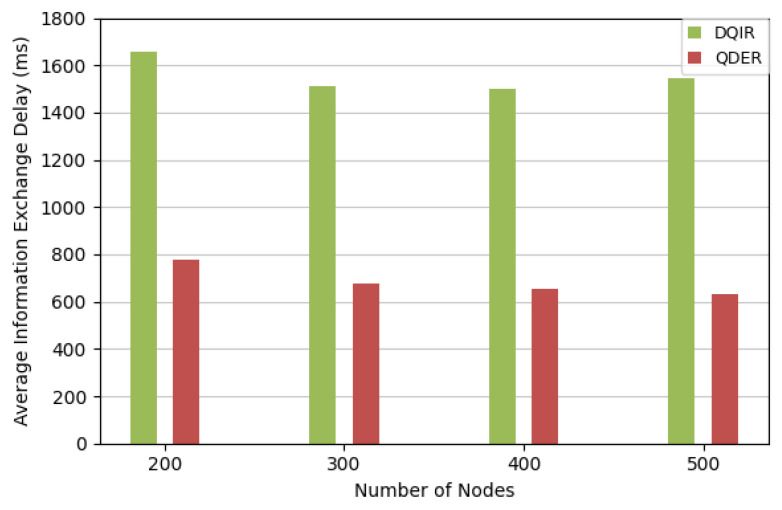
Average information exchange delay.

Figure 11 shows a comparison of the average information exchange delay $TDC_{ave}$ between the QDER and DQIR. In QDER, each node maintains a local Q-table and makes routing decisions based on its own information and that of neighboring nodes. Therefore, information exchange occurs only between adjacent nodes during the forwarding process. In contrast, DQIR adopts a centralized training mechanism, where the DQN node collects information from all nodes in the network. As a result, the amount of information exchanged in the centralized scheme is significantly larger. Consequently, the distributed design in QDER requires less information exchange and leads to shorter information exchange delay. The results shows that compared with DQIR, the QDER remarkably reduces the information exchange delay by over 50%.

**Figure 12:**
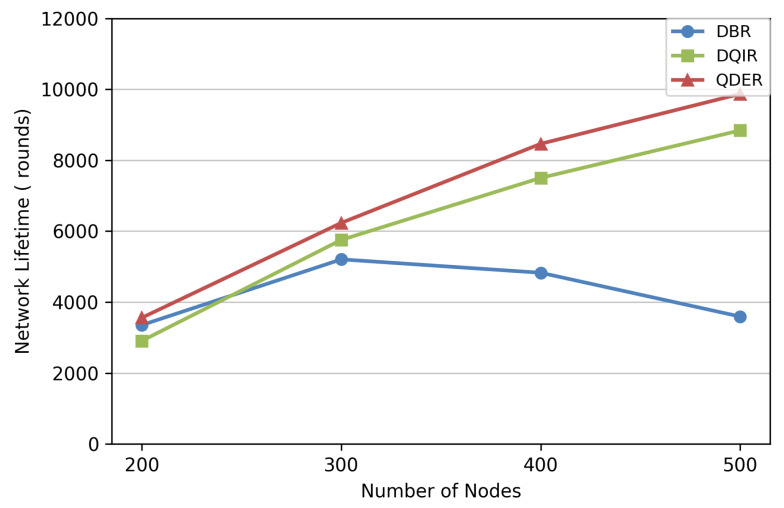
Network lifetime.

The network lifetimes are evaluated in Figure 12. It can be observed that QDER has a longer network lifetime than the other protocols. The benefit of QDER on the network lifetime becomes increasingly significant as the number of nodes increases. In the sparse deployment, the topology is relatively simple and energy consumption is inherently uniform, so that the performance gap among routing protocols is negligible. By contrast, as the topology becomes more complex, some nodes may consume their energy more rapidly because of frequent packet forwarding, which will shorten the total network lifetime. For this case, the QDER jointly considering residual energy and depth to select more efficient routing, thereby extending the network lifetime.

### 5.3. Performance Evaluation with Link Quality

To evaluate the impact of introducing link quality into the routing decision, simulations are performed in this section based on the Thorp’s channel transmission model, which accounts for physical-layer channel conditions. We use the reward function defined in Equation (9), considering link quality to enhance the robustness of the proposed algorithm against transmission noise. The improved version of the algorithm is referred to as IQDER (Improved QDER) in the simulation. The performance of BER, energy consumption, and network lifetime is evaluated.

**Figure 13:**
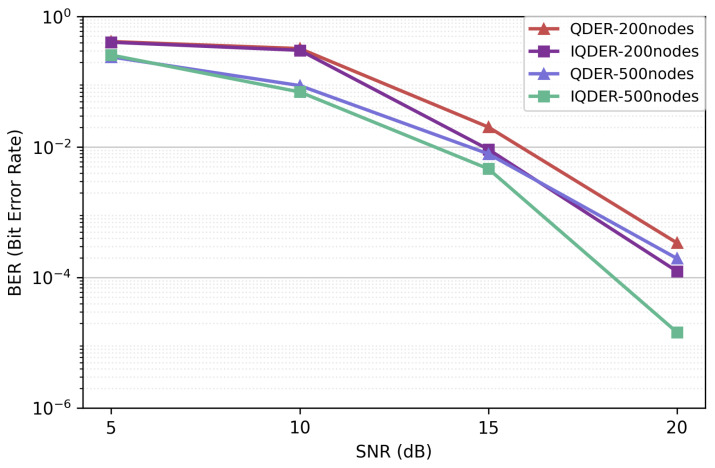
BER performance.

As shown in Figure 13, the BER from the source node to the sink decreases as the SNR increases. Under the same number of network nodes, the IQDER achieves a lower BER than that of QDER, demonstrating the effectiveness of considering link quality into the routing decision process. For a given SNR, the BER decreases as the number of network nodes increases. This trend can be explained by the fact that a denser network provides more candidate routing paths between the source and the sink. With a larger node number, the algorithms are more likely to converge to routes with fewer hops. Since shorter end-to-end paths leads to less transmission errors, the BER is correspondingly reduced.

**Figure 14:**
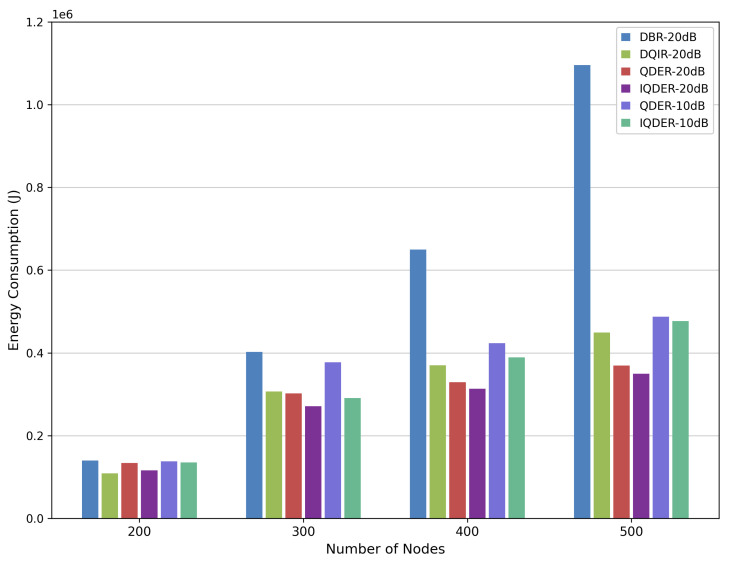
Energy consumption.

**Figure 15:**
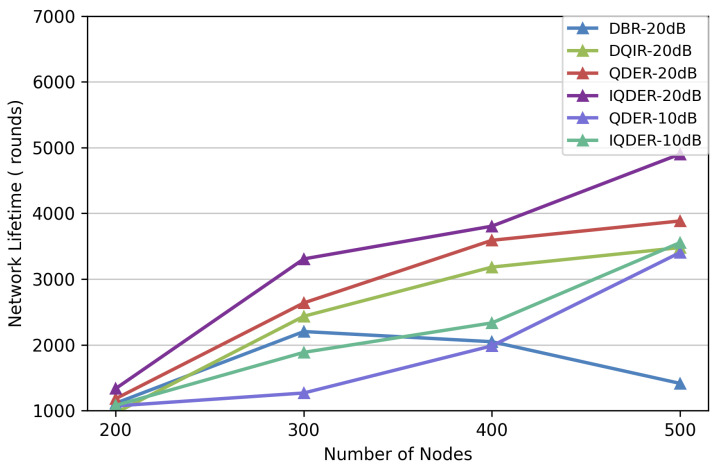
Network lifetime with link quality.

We also evaluate network energy consumption and network lifetime under SNR values of 10 dB and 20 dB. As shown in Figure 14 and Figure 15, the performance of IQDER is better than that of the other algorithms, particularly as the number of nodes increases. Benefiting from distributed processing, QDER and IQDER require less information exchange and fewer data retransmissions during packet forwarding, thus achieving higher energy efficiency and a longer network lifetime compared with both DBR and DQIR.

Furthermore, IQDER shows additional improvements over QDER because it uses the received SNR in the routing decision. By considering link quality during next-hop selection, nodes tend to choose links with more reliable transmission conditions, which reduces packet loss and unnecessary retransmissions. As a result, energy consumption is further reduced and the network lifetime is extended. These results demonstrate that introducing physical-layer information into the routing design is an effective approach for improving network performance in underwater acoustic sensor networks.

## 6. Conclusions

This paper proposes a QDER protocol for underwater acoustic sensor networks. The routing problem is formulated as an MDP, where each node maintains a local Q-table and makes routing decisions based on the depth and residual energy of neighboring nodes, enabling balanced energy consumption and efficient routing. In addition, link quality is taken into account in the reward function so that the impact of the physical channel is considered during relay node selection. Simulation results show that QDER achieves higher energy efficiency than DQIR and reduces information exchange delay by more than 50%, while also extending the network lifetime compared with DQIR and DBR under large-scale node deployments. Considering channel attenuation and noise, the proposed IQDER further improves energy efficiency and network lifetime and demonstrates robustness under different SNR conditions.

## Figures and Tables

**Table 1:** Simulation parameters.

UASNs Parameter	Symbol	Value
Number of relay nodes	$n_{f}$	200/300/400/500
Node initial energy (J)	$e_{ini}$	5000
Transmit power (*w*)	$p_{s}$	2
Received power (*w*)	$p_{r}$	1
Idle power (*w*)	$p_{w}$	0.01
Maximum transmission range (m)	*D*	100
Residual energy weight coefficient	$\beta_{1}$	0.8
Depth weight coefficient	$\beta_{2}$	0.2

**Table 2:** Symbol definitions.

Symbol	Definition	Symbol	Definition
*E*	total energy consumption	$T_{s_{i}}$	single packet transmitting time of $N_{f_{i}}$
$E_{N_{f_{i}}}$	energy consumption of $N_{f_{i}}$	$T_{r_{i}}$	single packet receiving time of $N_{f_{i}}$
$E_{R}$	energy consumption ratio	$T_{a_{i}}$	total running time of $N_{f_{i}}$
$E_{v}$	energy consumption variance	$T_{d_{i,j}}$	information exchange time between $N_{f_{i}}$ and $N_{f_{j}}$
$E_{ave}$	average energy consumption for network	$TDC_{m}$	information exchange delay for *m*-th data packet
$M_{data}$	number of data packets	$TDC_{ave}$	average information exchange delay

## Data Availability

The data presented in this study are available on request from the corresponding author.
